# Navigating the “frontal lobe paradox”: integrating Real-Life Tasks (RLTs) approach into neuropsychological evaluations

**DOI:** 10.3389/fpsyg.2024.1394483

**Published:** 2024-05-07

**Authors:** Odelia Elkana

**Affiliations:** ^1^Behavioral Sciences, Academic College of Tel Aviv-Yaffo, Tel Aviv, Israel; ^2^The National Institute of Neuropsychological Rehabilitation, Tel Aviv, Israel

**Keywords:** frontal lobe paradox, frontal lobe dysfunction, executive function, dysexecutive syndrome, prefrontal lobe, neuropsychological assessment, Real-Life Task (RLT), RLT

## Introduction

Individuals with frontal lobe damage often exhibit proficiency in interviews and standardized assessment tests, while experiencing significant impairments in their daily functioning—an intriguing phenomenon known as the “frontal lobe paradox” (Stuss and Benson, 1984; Burgess et al., 2006, 2009; Worthington, 2019; Fisher-Hicks et al., 2021; Newstead et al., 2022).

Within the subset of patients with prefrontal cortex (PFC) damage, there is a notable competence observed during clinical interviews and traditional assessments. However, these individuals frequently demonstrate substantial limitations in adaptive functioning, contributing to the complexity of the “frontal lobe paradox” (Stuss and Benson, 1984; Walsh, 1985) or the “knowing-doing dissociation” (Teuber, 1964; Luria, 1980). This not only challenges the clinician's understanding but also places the neuropsychologist in a predicament, as they must grapple with explaining this discrepancy or, in extreme cases, ignore test results that do not align with their diagnosis conclusion. Moreover, failure to address this discrepancy during standardized neuropsychological assessments can have profound consequences for patients, potentially impeding their access to necessary care and supervision, and even exposing them to risks (Fisher-Hicks et al., 2021).

Wood and Bigler (2017) emphasize the significance of conducting comprehensive interviews with individuals who have direct insight into the person's real-world behavior over time to avoid forming misguided opinions solely based on test performance. Burgess et al. (2009) further note that these patients may articulate plans and recall their actions but ultimately struggle to execute intended tasks.

Although many neuropsychologists are familiar with the “frontal lobe paradox”, it is common to face challenges in identifying such impairment solely based on standardized test results in typical clinical settings. George and Gilbert (2018) discuss these challenges in relation to the “frontal lobe paradox”, addressing the limitations of existing assessment tools and providing insights into the factors contributing to successful performance on standard tests.

To address the challenges posed by the “frontal lobe paradox” (Burgess et al., 2006; Wood and Bigler, 2017; Worthington, 2019; Fisher-Hicks et al., 2021) and ensure comprehensive and valid neuropsychological evaluations, it is imperative to incorporate ecological validity assessment into the assessment process (Goldstein and Scheerer, 1941; Burgess et al., 2006; Fisher-Hicks et al., 2021). Such assessment involves the evaluation of individuals' abilities in real-world contexts, providing valuable insights into their functional abilities and adaptive behaviors in everyday life settings. By supplementing traditional standardized tests with ecological validity measures, clinicians can gain a more holistic understanding of patients' cognitive functioning and identify discrepancies between performance in controlled testing environments and real-life situations. This integrative approach allows for a more nuanced assessment of executive functioning and self-initiation, particularly in individuals with frontal lobe damage who may demonstrate a disconnect between their performance on standardized tests and their functional abilities in daily life.

The following sections will explore various domains of executive functions along with corresponding RLT examples. This is intended to stimulate further consideration rather than presenting a definitive protocol for integrating RLT into neuropsychological assessment.

## Proposed evaluation approach for frontal lobe dysfunction—integrating “Real-Life Tasks” (RLT)

### Task initiation and execution of goal-directed behaviors

Executive functioning deficits, particularly in task initiation, are commonly observed in individuals with damage to the prefrontal cortex (PFC) (Stuss and Benson, 1984). Despite intact cognitive abilities measured by traditional neuropsychological tests (Lezak et al., 2012; Goldstein et al., 2013), these individuals often struggle with initiating and executing goal-directed behaviors. This challenge becomes more pronounced in unstructured tasks, where the individual must rely on internal cues and self-initiation to begin and complete activities. Therefore, assessing task initiation abilities within the context of daily life activities is crucial, as it provides valuable insights into individuals' functional capacities and adaptive behaviors.

**RLT Example:** Present the participant with unstructured tasks (e.g., making a coffee, organizing a desk). Instruct them to start each task without specific guidance. Assess their ability to initiate tasks without external cues and prompts.

To further illustrate, let's delve into the coffee-making example. The participant is asked to make a cup of coffee. Initially, the clinician observes whether the participant asks for directions or clarification, such as the location of the kitchen or where to find the necessary utensils. Then, as the participant progresses through the task, the clinician observes how they navigate each step of the process, from boiling water to selecting and adding sugar or a sugar substitute, choosing and adding milk or a milk substitute, and finally, locating a spoon to mix the coffee. The participant's ability to initiate each step of the task without external guidance is evaluated, along with their overall proficiency in completing the task independently.

### Behavioral organization in non-routine situations

Individuals with frontal lobe damage often struggle with planning, organizing, and adapting to novel or complex tasks, indicative of executive functioning deficits (Gioia et al., 1996; Burgess et al., 2006; Lezak et al., 2012).

**RLT Example:** Create a scenario requiring the participant to plan a social gathering such as a dinner party or a family barbecue given specific event details such as guest count, dietary restrictions, and budget constraints. They must then devise a detailed plan, covering menu selection, ingredient shopping, meal preparation, and venue setup.

During the task, the clinician observes how the participant organizes and prioritizes tasks, allocates resources (time, money), and handles potential challenges. The participants' written plan provides insight into their organizational strategies.

To further evaluate behavioral organization skills, the clinician can assess:

- **Menu planning**: does the participant create a balanced menu considering guest preferences and dietary needs, along with cost-effectiveness and ease of preparation?
- **Budget management**: how effectively does the participant allocate the budget to different event aspects, staying within constraints?
- **Time management**: does the participant develop a timeline for tasks, understanding the time needed for each activity?
- **Problem-solving skills**: how does the participant handle unexpected challenges, demonstrating flexibility and adaptability in their planning?

### Insight and compensatory strategies

Individuals with frontal lobe damage commonly exhibit insight deficits, lacking awareness of their cognitive impairments and their impact on daily functioning (Stuss and Benson, 1984; Scott and Schoenberg, 2011). This hinders their ability to employ effective compensatory strategies.

**RLT Example:** Ask the participant to reflect on a situation where they faced a cognitive challenge, such as managing multiple tasks simultaneously in a busy workplace environment, such as a restaurant kitchen or a retail store during a sale event and adapting to unexpected changes. The participant is instructed to imagine themselves in this scenario and describe how they would handle the situation. Inquire about their awareness of the difficulty and strategies employed to cope. Evaluate their ability to recognize and address cognitive impairments.

During the scenario, the clinician observes the participant's ability to recognize and address cognitive challenges in real-time. The participant may encounter unexpected changes or obstacles, such as a sudden influx of customers or equipment malfunctions. They are asked to verbalize their thoughts and actions as they navigate through the scenario, providing insights into their problem-solving strategies and coping mechanisms.

Key Aspects to Assess:

- **Awareness of cognitive challenges**: does the participant demonstrate an awareness of the cognitive demands of the scenario, such as the need to multitask and prioritize tasks effectively? Do they recognize the potential challenges they may encounter, such as managing time constraints or dealing with unexpected events?
- **Employed strategies**: what strategies do the participant employ to cope with cognitive challenges and maintain performance? Do they demonstrate effective organization, time management, and decision-making skills in response to the demands of the scenario?
- **Flexibility and adaptability**: how does the participant respond to unexpected changes or disruptions in the scenario? Do they demonstrate flexibility and adaptability in adjusting their strategies and priorities to address new challenges as they arise?
- **Insight into cognitive impairments**: does the participant acknowledge any difficulties or limitations they experience during the scenario? Are they able to identify specific cognitive impairments or challenges they face, such as memory lapses or attention deficits?

### Rule maintenance and cognitive flexibility

Frontal lobe damage can lead to impairments in rule maintenance and cognitive flexibility (Shallice and Burgess, 1996; Diamond, 2006). Individuals with such damage may struggle to maintain rules and adapt their behavior according to changing task demands, indicating deficits in executive functioning.

**RLT Example**: Modified “Uno” Rule Maintenance Task

Objective: Assess the participant's ability to maintain rules and adapt to changes in a modified version of the card game “Uno”.

Instructions:

- Set up the game by shuffling the deck of Uno cards and dealing seven cards to each player, including the participant.
- Explain the basic rules of Uno and play several rounds.
- Then, change a rule or two in the game.
- Play several rounds of the modified Uno with the participant, ensuring they adhere to the rules and demonstrate understanding.
- Introduce variations and rule changes throughout the game to assess adaptability.
- Observe the participant's ability to maintain focus, follow evolving rules, and adapt strategy.
- Record any difficulties experienced in maintaining rules or adapting to modifications.

This modified Uno task provides a structured yet flexible assessment of rule maintenance and cognitive flexibility, mimicking real-life situations where individuals must adhere to rules and adjust their behavior accordingly.

### Social cognition

Impairments in social cognition are frequently observed in individuals with frontal lobe damage (Knight and Grabowecky, 1995; Amodio and Frith, 2006). These individuals may struggle with interpreting social cues, understanding others' perspectives, and regulating their social behavior, reflecting deficits in social cognition.

**RLT Example:** Present a social scenario (e.g., a video clip or written description) and ask the participant to interpret emotions, intentions, and social dynamics.

An example can be a scene from the movie ‘Forrest Gump', where Forrest attends a social gathering at his friend Lieutenant Dan's house. The subtext in this scene revolves around Forrest's innocence and straightforwardness contrasted with the complexity of social interactions happening around him.

Several aspects warrant attention:

- **Emotion interpretation**: assess the participant's understanding of the emotions experienced by the characters in the scenario. This involves identifying emotions accurately based on verbal and nonverbal cues. The clinician can ask the patients: What emotions do you think Forrest and the other characters are experiencing during the interaction?

- **Intention recognition**: evaluate the participant's ability to discern the intentions or motivations behind the words and actions of the characters. This involves inferring underlying motives from observable behaviors. The clinician can ask the patients: What do you believe are their intentions or motivations behind their words and actions?
- **Social dynamics**: analyze the participant's interpretation of the social dynamics between the characters. Determine whether they recognize the nature of the relationships, such as whether they are friendly, competitive, supportive, or indifferent. The clinician can ask the patients: How would you interpret the dynamics between Forrest and the other guests? Are they friendly, competitive, supportive, or indifferent?
- **Response to social cues**: consider how the participant would respond if they were in the situation depicted in the scenario. Assess their ability to appropriately react to social cues and interactions, taking into account their understanding of the context and their own social norms. The clinician can ask the patients: If you were Forrest in this situation, how would you respond to the various social cues and interactions?

Overall, attention should be given to the participant's comprehension of social nuances, their ability to accurately interpret social situations, and their capacity to respond appropriately to social cues, reflecting their social cognition abilities.

## Discussion and conclusion

This opinion article aims to offer a broad trajectory for future explorations concerning the nuanced assessment of executive functioning deficits and their implications for RLT performance. Through the enhancement of assessment protocols and the inclusion of thorough observations encompassing RLTs of initiation, execution, organizational planning, social cognition, and insight—clinicians can acquire deeper insights into the functional capabilities of individuals with prefrontal cortex damage. Additionally, the classification of specific types of mistakes made during task completion could inform targeted interventions tailored to address identified deficits. Overall, the integration of RLTs into neuropsychological evaluation holds promise for enhancing the accuracy, validity, and clinical utility of assessments for individuals with executive functioning impairments.

These RLT examples aim to evaluate various ecological dimensions of executive functioning associated with frontal lobe damage. However, it's essential to acknowledge that these examples represent only a subset of the challenges individuals with frontal lobe damage may face (Duncan, 1986; Delis et al., 2001; Stuss and Alexander, 2007; McCloskey et al., 2009; Damasio et al., 2011; Worthington, 2012; Otero and Barker, 2014). Additional domains, such as decision-making, can also be incorporated into RLT protocols, highlighting the need for ongoing development and refinement in this area.

It is crucial to customize tasks according to individual abilities and consider cultural and contextual factors during test administration.

This RLT approach might challenge the conventional practices of neuropsychologists, given that our professional training often centers on structured and standardized tests that yield normalized scores. However, in light of the “frontal lobe paradox”, it becomes imperative to step outside the traditional framework and gather ecological data on the patient's actual executive abilities.

While concerns may arise regarding the lack of normative data for the proposed RLTs, their effectiveness is assessed based on success or failure, including partial success with specific types of errors, without relying on comparisons to established norms or standards. In other words, whether the tasks are successful or not can be judged based on their specific objectives and criteria, rather than comparing them to how others perform.

Presenting the results of these tasks, such as “X RLTs were administered to assess executive functioning, and patients failed to fulfill them successfully in a Y/X ratio… The type of errors/difficulties included…” can provide valuable additional insights in the report, particularly when complemented with etiology data and findings from brain imaging (MRI/CT), as well as outcomes from tailored tests to confirm or reject the presence of executive dysfunction.

In conclusion, the “frontal lobe paradox” presents a significant challenge in neuropsychological assessments, highlighting the need for a comprehensive approach that goes beyond traditional standardized tests. The incorporation of ecological validity assessment in the form of RLT, as proposed in this manuscript, may offers a potentially useful approach for addressing this paradox by providing a more nuanced understanding of individuals' cognitive functioning in real-world contexts.

Future research should focus on:

- Developing specific protocols for Real-Life Tasks (RLTs) and validating their effectiveness in assessing executive functioning deficits in individuals with frontal lobe damage.
- Empirically evaluating the efficacy of incorporating RLTs into neuropsychological evaluations for individuals with frontal lobe damage, comparing their outcomes with those of traditional standardized tests.
- Investigating the impact of ecological assessments, specifically RLTs, on treatment planning and outcomes for individuals with frontal lobe damage.
- Assessing the feasibility of implementing RLTs in routine clinical practice and evaluating their effectiveness in improving patient care and outcomes.

By systematically evaluating the benefits and limitations of incorporating RLTs, researchers and clinicians can better understand their role in addressing the real-world needs of individuals with frontal lobe damage. Ultimately, this approach can contribute to the refinement and optimization of neuropsychological assessment protocols, leading to improved assessment, care, and outcomes for this patient population.

This opinion manuscript serves as a call for further contemplation, research, and development in the context of integrating RLT into the standard neuropsychological assessment.

## Author contributions

OE: Conceptualization, Writing – original draft, Writing – review & editing.
